# From “performance competition arena” to “psychological exemption zone”: psychological safety mechanisms in reverse mobility

**DOI:** 10.3389/fpsyg.2026.1785539

**Published:** 2026-06-16

**Authors:** Peitao Du, Tingting Liu, Xujin Xian

**Affiliations:** 1 Al-Farabi Kazakh National University, Almaty, Kazakhstan; 2 The Catholic University of Korea, Bucheon, Republic of Korea

**Keywords:** constructivist grounded theory, psychological exemption zone, psychological safety, reverse mobility, setting-seeking, workplace environment

## Abstract

In contemporary China, increasing numbers of Ph.D.-holding corporate professionals and new-generation doctoral-stage participants (Ph.D. candidates or Ph.D. graduates within 1–2 years) prioritize university faculty or research posts (“reverse mobility”). Prior accounts have focused mainly on economic and institutional push–pull forces; consequently, the psychological and meaning-making processes driving this shift remain under-specified. Accordingly, reverse mobility is conceptualized as a form of setting-seeking, whereby individuals pursue work settings perceived as more restorative, predictable, and boundary-controllable. Using constructivist grounded theory, 47 participants (25 transitioners and 22 new-generation doctoral-stage participants) were interviewed, and iterative open–axial–selective coding with constant comparison and triangulation was conducted until theoretical saturation. Participants depict marketized workplaces as a “performance competition arena” characterized by KPI-driven value alienation, chronic exhaustion, and anticipatory insecurity. When perceived risk crosses a subjective threshold, psychological safety needs become salient and shift decisions from escape toward shelter seeking. Universities are then symbolically constructed as a “psychological exemption zone,” namely a perceived work setting that can reduce exposure to marketized performance risks and help restore recovery time, predictability, and control over work–life boundaries. This construction is supported by institutional shelter, time sovereignty, meaningful work, and community belonging. Participants then enact identity reconstruction, narrative management, and boundary work to turn this imagined safer setting into an actual career move and a subjective sense of relocation from one work world to another. Transitioners emphasize restoration after accumulated strain, whereas new-generation doctoral-stage participants foreground preventive risk avoidance. This study develops a dynamic process model integrating need-based mechanisms with risk perceptions, extending reverse-mobility research by foregrounding psychological safety and defensive career strategies.

## 1 Introduction

In contemporary China, a salient trend of “reverse mobility” has emerged among Ph.D.-holding corporate professionals and new-generation doctoral-stage participants: they increasingly prioritize university faculty positions or research posts–particularly in teaching-oriented or less research-intensive higher-education institutions–as their preferred career choice (Li and Horta, 2024; Schauer, 2025; Tang et al., 2025; Yin, 2025; YouTu Study Abroad, 2025). Media narratives often portray this phenomenon as a pragmatic pursuit of “secure tenure” and “work–life balance,” while scholarly debates have largely remained confined to economically rational, institutionally oriented push–pull frameworks (Kallio et al., 2024; Li et al., 2024; Sullivan and Al Ariss, 2021; Zhai et al., 2021). Yet these accounts leave unresolved a central issue: When individuals face consequential career decisions, What deeper psychological motives and affective logics lead them to forgo high-income potential or mainstream career trajectories (Akkermans et al., 2024; De Vos et al., 2020; Hirschi and Koen, 2021)? This issue matters because recent career scholarship increasingly treats mobility decisions as dynamic processes shaped by career self-management, sustainable-career concerns, risk appraisal, and meaning-making rather than as one-time responses to external incentives (Akkermans et al., 2024; De Vos et al., 2020; Hirschi and Koen, 2021; Sullivan and Al Ariss, 2021). Existing research has paid limited attention to meaning-making, shifts in psychological needs, and identity reconstruction during career transitions, leaving a critical gap in knowledge. In this study, “enterprise transitioners” refers to Ph.D. holders who previously held full-time professional or managerial roles in marketized firms and subsequently moved into academia. “New-generation doctoral-stage participants” refers to final-year Ph.D. candidates or Ph.D. graduates within 1–2 years after graduation who are considering or pursuing academic employment.

To address this gap, this study adopts an integrative perspective drawing on educational psychology and career development theories, moving beyond conventional utilitarian explanations. The bridge from the phenomenon to the research question lies in treating reverse mobility not only as movement across sectors but also as a setting-seeking process through which individuals search for recovery, predictability, meaning, and boundary control under perceived occupational risk. The core theoretical construct of a “psychological exemption zone” is proposed to capture how participants imagine and evaluate a university work setting as a safer alternative to marketized workplaces. More specifically, it describes a subjectively constructed career-setting representation that is expected to reduce exposure to performance risks and restore three valued conditions: restorability, predictability, and boundary controllability. This concept helps examine how individuals perceive and construct certain university settings as symbolic spaces that provide emotional shelter, restore autonomy, and enable the recovery of meaning (Blustein et al., 2023; Edmondson and Bransby, 2023; Gagné et al., 2022; Ryan and Deci, 2020; Van den Broeck et al., 2021). Accordingly, the study asks three connected research questions: (1) How do participants construct marketized workplaces as “performance competition arenas”? (2) How do they imagine and evaluate universities as “psychological exemption zones”? (3) Through what identity, narrative, and boundary-work strategies is this imagined safer setting translated into career action? “Restorability” refers to the perceived potential of a work setting to enable psychological recovery from prior strain. “Boundary controllability” indicates the extent to which individuals can control the boundaries between work and non-work life (e.g., time, space, and mental separation). “Psychological migration” denotes participants’ felt shift from one work world to another–from a corporate identity organized around performance competition to an academic identity associated with protected autonomy and meaning. This shift may begin before the formal job transition is fully completed. Using a constructivist grounded theory approach, in-depth interviews were conducted with 25 “transitioners” who moved from enterprises into academia and 22 “new-generation” doctoral-stage participants who chose academia directly. These accounts were further triangulated with textual materials to develop, from the ground up, a theoretical model of the psychological mechanisms underlying this phenomenon.

This study contributes a systematic account of psychological-safety seeking and defensive career strategies as central drivers of “reverse mobility.” By integrating self-determination theory with risk perception theory, an integrative analytical framework is developed to clarify how perceived psychological risks and need frustration in marketized work environments can reorient career decisions toward settings expected to be more predictable and controllable (Ally and Hagger, 2024; Gagné et al., 2022; Rudolph et al., 2021; Ryan and Deci, 2020; Van den Broeck et al., 2021; Zheng and Yan, 2024). Practically, the findings offer empirically grounded implications for talent management in universities, for enterprises’ reflection on organizational health, and for individuals’ more reflective career planning. The following sections elaborate the theoretical background, methodological design, and key findings of the study.

## 2 Literature review

### 2.1 Prior research on reverse mobility

Research on the career transition of “high-paying Ph.D.-holding corporate professionals/doctoral-stage participants moving ‘in reverse’ toward universities” has primarily advanced along three explanatory pathways. First, scholars emphasize structural opportunities and institutional benefits, such as tenure-like staffing systems, hukou (China’s household registration system)-related advantages, and welfare protections (Li et al., 2024; Wang and Wang, 2024; Zhai et al., 2021). Second, studies draw on a human-capital and career-development logic, focusing on person–job fit, career self-management, and promotion pathways (Akkermans et al., 2024; De Vos et al., 2020; Hirschi and Koen, 2021; Spurk et al., 2019; Sullivan and Al Ariss, 2021). Third, research highlights working conditions and organizational governance, including working-hour regimes, performance pressure, and managerial practices (Bakker et al., 2023; Chen et al., 2023; Matsuda and Kikutani, 2024).

Although these streams clarify the institutional context and organizational constraints shaping mobility, they share a key limitation: they tend to reduce career choice to a static response to external incentives and changing conditions (Kallio et al., 2024; Li et al., 2024; Sullivan and Al Ariss, 2021; Zhai et al., 2021). As a result, they struggle to explain why individuals make sharply different–and enduring–career decisions under similar structural circumstances. Recent organizational psychology and career research argue that career decision-making is better understood as a dynamic trade-off between psychological risks and psychological resources under labor-market uncertainty (Akkermans et al., 2024; De Vos et al., 2020; Hirschi and Koen, 2021; Rudolph et al., 2021). When work settings continuously erode psychological safety, deplete self-regulatory resources, and undermine work meaning, exit may function as an active psychological defense strategy (Bakker et al., 2023; Blustein et al., 2023; Edmondson and Bransby, 2023). However, existing research has not sufficiently theorized this psychological transformation process. In particular, it remains unclear how deteriorating work environments translate into impaired psychological safety and accumulated burnout, and how these experiences subsequently reshape meaning appraisals and propel career redirection. Accordingly, the chained mechanism linking psychological safety, burnout, and meaning in career choice constitutes a critical gap that warrants systematic investigation.

### 2.2 Psychological mechanisms of career choice

An expanding body of research conceptualizes career choice as a chained mechanism that connects psychological safety, burnout, and work meaning (Bakker et al., 2023; Blustein et al., 2023; Edmondson and Bransby, 2023; Newman et al., 2017). In this view, sustained threats to psychological safety foster burnout, which then reshapes individuals’ evaluations of work meaning and career direction.

A substantial body of research links diminished psychological safety directly to increased turnover intention. Psychological safety, widely viewed as a contextual resource that enables employees to speak up and experiment without fear of reprisal (Edmondson and Bransby, 2023; Newman et al., 2017), is often eroded in high-pressure organizations by punitive accountability, excessive performance comparison, and opaque evaluation systems (Bakker et al., 2023; Shao et al., 2022). Evidence from healthcare settings similarly indicates that leader-related psychological safety predicts job satisfaction and turnover intention (Shao et al., 2022). These findings imply that when private-sector workplaces exhibit a “high-risk interaction–low-support” climate, employees may increasingly imagine universities as environments where such psychological threats are less pronounced.

Parallel findings emerge from research on burnout, particularly within China’s demanding “996” work culture. Job demands–resources theory suggests that excessive demands and insufficient resources contribute to exhaustion and disengagement (Bakker et al., 2023), while research on Chinese Gen Z employees indicates that “996” schedules (9 a.m. to 9 p.m., 6 days per week) are positively associated with work overload and burnout, which in turn is strongly related to psychological distress (Chen et al., 2023; Matsuda and Kikutani, 2024). From this perspective, “reverse mobility” may represent not a simple preference for ease, but rather an attempt to avoid long-term emotional exhaustion and psychosomatic risk, and to restore basic psychological functioning.

Finally, the intertwined concepts of decent work, meaningful work, and sustainable careers provide another crucial lens. Recent scholarship has shifted from an exclusive emphasis on “calling” to a combined “meaning–decent work” framework (Blustein et al., 2023), and sustainable-career research emphasizes the long-term alignment of productivity, health, and happiness (De Vos et al., 2020). When decent work conditions are absent–such as predictable working hours, fair compensation, dignity, and safety–narratives of meaning may ironically become vehicles for self-exploitation. For many participants, the appeal of university positions may thus derive not only from imagined meaning (e.g., teaching and knowledge creation), but also from the prospect of more predictable work rhythms and institutional protections (Blustein et al., 2023; De Vos et al., 2020; Ryan and Deci, 2020).

### 2.3 Self-determination theory and risk perception

Self-determination theory (SDT) posits three basic psychological needs–autonomy, competence, and relatedness (Ryan and Deci, 2020). Recent workplace reviews and meta-analytic evidence indicate that organizational support for these needs systematically shapes motivational quality, stress responses, work motivation, and sustained engagement (Ally and Hagger, 2024; Gagné et al., 2022; Van den Broeck et al., 2021). In corporate settings, controlling management and short-cycle KPI systems can constrain autonomy. Competitive “involution” dynamics may also weaken relatedness support and render competence more fragile (Mulvey and Wright, 2022). By contrast, universities are often perceived as offering greater research and teaching autonomy, longer time horizons for professional development, and access to academic community resources. Such features may be interpreted as better satisfying SDT needs, thereby enabling a career narrative that moves “toward intrinsic motivation.”

In addition, post-pandemic risk perception appears to have reshaped career preference structures. Recent evidence from Chinese university students suggests that, under pandemic normalization, employment confusion, anxiety, and preferences for stability have intensified and meaningfully influence job intentions (Zheng and Yan, 2024). Broader career research similarly suggests that uncertainty, career shocks, and sustainable-career concerns can alter how individuals evaluate long-term career viability (Akkermans et al., 2024; De Vos et al., 2020; Rudolph et al., 2021). This line of work indicates that career decisions increasingly reflect efforts to avoid uncertainty and health-related risks. Between a private-sector option characterized by “high pay–high risk–high depletion” and a university option associated with “moderate returns–greater predictability,” individuals may rationally gravitate toward the latter to reduce long-term psychological risk.

### 2.4 Defining the “psychological exemption zone” and this study’s contribution

Building on these developments, this study proposes the “psychological exemption zone” as a core analytical concept. It refers to an occupational and organizational space that individuals actively seek or construct to achieve temporary exemption from systemic psychological risks embedded in marketized career environments (i.e., “performance competition arenas”). Such risks include impaired psychological safety, chronic burnout, erosion of meaning, and identity alienation (Bakker et al., 2023; Blustein et al., 2023; Edmondson and Bransby, 2023; Newman et al., 2017). Empirically, universities are perceived by some groups as alternative settings that offer greater psychological safety, more accessible meaning, and stronger satisfaction of autonomy–competence–relatedness needs (Blustein et al., 2023; Edmondson and Bransby, 2023; Gagné et al., 2022; Ryan and Deci, 2020; Van den Broeck et al., 2021). Adopting a place-based lens, this study further argues that career decisions also reflect evaluations of work settings through salient place cues and perceived affordances (e.g., predictability, boundary controllability, and socio-spatial climate). Theoretically, the concept links micro-level psychological mechanisms (safety, meaning, and basic needs) with macro-level risk perception (uncertainty, health, and career sustainability) (De Vos et al., 2020; Rudolph et al., 2021; Zheng and Yan, 2024). In doing so, it provides a testable explanatory framework for understanding differentiated choices under similar structural conditions. From this perspective, universities are attractive not only as organizations but also as places perceived to support restoration, preserve daily rhythms, and enable identity-consistent meaning practices.

## 3 Data sources and research methods

### 3.1 Research design

To examine the complexity, dynamism, and contextual contingency of psychological motives during career transitions, this study adopts a qualitative research paradigm and employs constructivist grounded theory as its primary methodological guide (Bobbink and Larkin, 2024; Busetto et al., 2020). This choice is justified on three grounds. First, the study addresses “how” and “why” questions–how individuals construct the meaning of their mobility and why universities are perceived as a “psychological exemption zone.” Qualitative research is well suited to topics centered on meaning interpretation and process mechanisms (Corbin and Strauss, 2014). Second, existing theoretical frameworks provide limited explanatory leverage for this phenomenon. Accordingly, the study requires bottom-up theory generation and/or refinement of extant concepts from empirical data, which lies at the heart of grounded theory (Charmaz, 2014). Third, the constructivist stance recognizes knowledge as co-produced through researcher–participant interaction, consistent with our emphasis on subjective experience and meaning-making.

The research design foregrounds comparison and theory building. Rather than offering a purely descriptive account, two analytically contrasting groups–“enterprise transitioners” and “new-generation doctoral-stage participants”–were purposively selected because they differ in career starting points, social timing, and risk perception. Constant comparison across these groups was conducted throughout the analytic process. For analytical clarity, this study defines “enterprise transitioners” as Ph.D. holders who previously held full-time professional/technical or managerial roles in marketized firms and subsequently moved into academia. “New-generation doctoral-stage participants” refer to final-year Ph.D. candidates or individuals within 1–2 years after graduation who are considering or pursuing entry into academic employment. The inclusion of the new-generation group was theoretically justified because this study treats reverse mobility not only as a completed occupational move but also as a pre-transition process of risk appraisal, meaning-making, and setting selection. In this design, enterprise transitioners illuminate a post-strain repair pathway, whereas doctoral-stage participants illuminate an anticipatory risk-avoidance pathway. Comparing these two groups makes it possible to examine whether experienced strain and anticipated insecurity converge on similar psychological safety needs and similar constructions of the university as a protective setting. This comparative strategy was intended to foster higher-level theoretical abstraction. For instance, it enabled examination of whether “experienced trauma” and “anticipated anxiety” lead to similar psychological needs and action strategies, thereby distilling more generalizable core categories (e.g., “psychological safety”). The study followed the iterative logic of classic grounded theory: data collection and analysis proceeded concurrently, early insights informed subsequent theoretical sampling, and the process continued until category saturation and an internally coherent theoretical model were achieved (Foley et al., 2021).

#### 3.1.1 Researcher positioning and reflexivity

The research team comprises two scholars with interdisciplinary training in education and psychology and long-term engagement with China’s competitive workplace culture and enterprise-to-university career pathways. To minimize the influence of prior assumptions on interpretation, reflexive memos were maintained throughout the study to record immediate reactions to the data and emerging theoretical connections. Regular cross-check discussions were also held to scrutinize and challenge interpretations. This sustained reflexive practice was designed to keep the analysis open to complexity and multiple voices, ensuring that the resulting theory was grounded in participants’ lived experiences rather than researchers’ preconceptions.

#### 3.1.2 Research context

This study is situated within China’s specific socioeconomic and discursive landscape. In recent years, terms such as “996,” “involution” (a spiraling process of intensified competition amid stalled mobility and diminishing returns), and “shang’an” (securing a stable, tenure-like position) have become widely salient. These discourses, together with the widespread implementation of “pre-tenure–tenure-track” reforms in universities, have contributed to a career ecology characterized by tension and uncertainty (Matsuda and Kikutani, 2024; Mulvey and Wright, 2022; Wang and Wang, 2024). We interpret participants’ narratives against this macro context to illuminate how broader social discourses are internalized and how they concretely shape career risk perceptions and imaginaries of the university as a “psychological exemption zone.”

### 3.2 Data collection

To enhance data richness and diversity and to enable triangulation (Santos et al., 2020; Schlunegger et al., 2024), data were collected through three primary channels between March and October 2025. Consistent with constructivist grounded theory, sampling followed the logic of theoretical sampling, such that recruitment was shaped by emerging analytic insights (Charmaz, 2014; Foley et al., 2021). Specifically, ongoing coding and constant comparison guided decisions about whom to sample next, with the goal of developing categories and elaborating their properties and dimensions. Initial recruitment purposively included two groups–“enterprise transitioners” and “new-generation doctoral-stage participants”–to initiate systematic comparison across contrasting risk experiences (experienced strain vs. anticipated anxiety). As initial insights and provisional categories began to take shape (e.g., the emergent category of “awakening psychological safety needs”), cases that could maximize conceptual variation and deepen category development were then sampled (e.g., adding transitioners from different industries and with varying urgency to resign) until theoretical saturation was reached (Naeem et al., 2024; Rahimi and Khatooni, 2024; Squire et al., 2024). Throughout, data collection and analysis were conducted iteratively and in parallel, integrating theoretical sampling with the constant comparative method. In addition, participants’ descriptions of work-setting cues and everyday artifacts were attended to as part of the comparative analysis.

#### 3.2.1 In-depth semi-structured interviews as the core data source

In-depth interviews served as the core strategy for generating participants’ career narratives, motivational accounts, and affective experiences. In line with theoretical sampling, both group delineation and sample size were determined analytically rather than pre-specified (Foley et al., 2021; Naeem et al., 2024; Rahimi and Khatooni, 2024; Squire et al., 2024). First, the two groups represent distinct career starting points and types of risk experience, providing an initial comparative frame. Second, the final sample sizes (25 vs. 22) were determined through iterative recruitment and analysis, guided by theoretical saturation: sampling within a group ceased when additional interviews no longer yielded new category-level insights or relationships but only further specified existing categories. The slight imbalance in group size reflects the greater heterogeneity of transitioners’ career histories, which required additional interviews to sufficiently elaborate category variation and reach saturation. The new-generation participants were therefore not treated as “quitters” in the narrow sense. Rather, they were selected to capture the anticipatory stage of reverse mobility, in which doctoral-stage individuals form risk perceptions, compare career settings, and construct universities as safer options before any completed corporate exit occurs. This sampling logic strengthens the study’s ability to connect completed transition with prospective career choice.

The semi-structured interview guide was initially developed and refined based on the research objectives and key concepts from the literature review, including psychological safety, self-determination theory, and career transition. To ensure clarity and relevance, the guide was pre-tested with two individuals (one former corporate professional and one doctoral student) who matched the participant profile but were not included in the formal sample. Their feedback led to minor adjustments in wording and question sequencing. Crucially, consistent with the constructivist grounded theory approach, the interview guide was treated as a flexible framework rather than a rigid protocol. As data collection and preliminary analysis progressed through theoretical sampling, emerging concepts (e.g., “boundary controllability,” “dignified exit”) informed the inclusion of new probes in subsequent interviews. The complete semi-structured interview protocol is included as Supplementary Material S2. This iterative process ensured that the interviews remained responsive to the participants’ lived experiences and the evolving theoretical focus of the study.

To capture heterogeneity relevant to category development, maximum variation was incorporated within key sampling dimensions. Gender was approximately balanced in both groups (transitioners: 13 men/12 women; new-generation: 11 men/11 women). Participants were drawn from major cities in eastern, central, and western China, and disciplinary backgrounds spanned STEM, business/management, and the humanities and social sciences. Destination institutions were primarily teaching-oriented or less research-intensive higher-education institutions (e.g., application-oriented universities and vocational colleges). In participants’ accounts, these settings were characterized by comparatively less intensive research-performance expectations and more predictable evaluation cycles than elite research universities. Importantly, these distinctions are reported as perceived features that informed participants’ setting evaluations, rather than as universal attributes of the university sector. This design increased variation along theoretically relevant dimensions, thereby strengthening the conceptual reach of emerging categories. Notably, the “average years of work/study” in Table 1 carries different analytic implications for the two groups. For transitioners, 8.5 years of corporate tenure suggests that “post-trauma exit” narratives often draw on cumulative exhaustion over time. For the new-generation group, doctoral-stage experience indicates sustained investment in the academic pathway; their “prospective risk avoidance” is grounded in greater familiarity with academic careers. Throughout this paper, including figures and tables, the label “new-generation” is used as shorthand for “new-generation doctoral-stage participants,” defined as final-year Ph.D. candidates or Ph.D. graduates within 1–2 years after graduation. The basic composition of interview participants is summarized in Table 1.

**TABLE 1:** In-depth semi-structured interviews (core data source).

Group	*N*	Average age	Mean years of work/study	Example source industries/fields	Example destination institution types
Enterprise transitioners	25	34.2	8.5 years in corporate roles	Internet/technology, finance/investment banking, advanced manufacturing	Application-oriented universities; research-oriented universities; vocational colleges
New-generation doctoral-stage participants	22	27.8	Final-year Ph.D. candidates or Ph.D. graduates within 1–2 years after graduation	STEM; humanities and social sciences; business/management	

“New-generation doctoral-stage participants” refers to final-year Ph.D. candidates or individuals within 1–2 years after graduation. “Enterprise transitioners” refers to Ph.D. holders who previously worked in marketized firms in professional/technical or managerial roles before moving to academia.

The interview guide covered key themes, including critical turning points in career trajectories; perceptions and evaluations of prior corporate work; the decision process and core considerations underlying the choice of university employment; expectations versus lived experiences in universities; and narratives of identity change. Interviews lasted 60–120 min and were audio-recorded with informed consent, transcribed verbatim, and compiled into a corpus of approximately 200,000 Chinese characters. Salient place cues and perceived affordances of work settings (e.g., predictability, rhythm stability, boundary controllability, and socio-spatial climate) were also probed because these cues shaped participants’ stress appraisals and recovery expectations.

#### 3.2.2 Online texts and public documents

As an auxiliary data source, online texts and public documents were used to situate participants’ accounts within broader discursive conditions and to support analytic comparison. More than 120 materials relevant to the study were systematically collected. These materials included media reports and commentaries on topics such as “talent returning to universities,” doctoral career choices, and “leaving big-tech firms”; high-engagement posts and comments from platforms such as Zhihu, Douban, and Xiaohongshu that reflect shared vocabularies (e.g., “going ashore,” a colloquial expression for securing a stable position; “lying flat,” referring to strategic effort reduction; and “high-stakes promotion policy”); and policy/statistical reports on university personnel reforms and youth employment. These sources were used as contextual and comparative materials to sharpen theoretical sensitivity and to triangulate emerging interpretations (Naeem et al., 2024; Rahimi and Khatooni, 2024; Santos et al., 2020; Schlunegger et al., 2024).

#### 3.2.3 Personal documents and artifacts

With participants’ voluntary consent, 18 personal documents were collected to capture reflections during career transition, including personal statements or research proposals for university applications; staged reflections posted on social media (e.g., WeChat Moments) about resignation or onboarding; and excerpts from private notes documenting work experiences. These “silent data” provided temporally immediate traces of emotions and reflections that complemented interview accounts. Analytically, they were incorporated into triangulation and constant comparison to refine category properties and to identify tensions that informed subsequent interview probes, thereby dynamically shaping the depth and direction of ongoing data collection.

All participants provided informed consent, and all materials were anonymized. The informed consent form is provided as Supplementary Material S1. Data collection and analysis proceeded in parallel. When the final five interviews produced no new major categories or relationships and primarily elaborated existing ones, theoretical saturation was judged to have been reached and further sampling was halted (Naeem et al., 2024; Rahimi and Khatooni, 2024; Squire et al., 2024; Staller, 2021).

### 3.3 Data analysis process and rigor strategies

Data analysis followed the iterative logic of constructivist grounded theory and was supported by NVivo 12 Plus for end-to-end coding, data management, and theory building, enhancing analytic rigor, systematicity, and auditability (Bobbink and Larkin, 2024; Charmaz, 2014). NVivo was used in three analytic moves aligned with the coding process. First, node creation and management (open coding): after importing interview transcripts, netnographic materials, and related texts, line-by-line coding was conducted and free nodes were created as initial codes (conceptual labels), while analytic memos were written to capture emerging insights. Second, node refinement and relationship mapping (axial coding): conceptually similar free nodes were clustered into tree nodes as categories, and the Relationship function was used to examine links among nodes (e.g., conditions, actions/strategies, and consequences), supporting the articulation of category structure. Third, theoretical integration and visualization (selective coding): the Model tool was used to visualize the evolving theoretical model–“stress/risk perception → defensive choice → boundary reconstruction”–and Query functions were employed to check the density of supporting evidence and the consistency of category linkages. The analytic process was recursive rather than linear, with constant comparison as the central strategy, ensuring that the emerging theory remained grounded in data while becoming progressively more abstract.

#### 3.3.1 Three-stage coding and theory generation

##### 3.3.1.1 Open coding

During open coding, line-by-line and incident-by-incident microanalysis was conducted for all 47 interview transcripts, netnographic texts, and observation notes. The analysis remained open so that codes and concepts could emerge from the data, rather than being imposed as *a priori* categories. Particular attention was paid to accounts that appeared passive on the surface yet implicitly conveyed agentic strategies and complex psychological work. This narrative pattern itself provided early analytic leverage for understanding how participants construct the university as a “psychological exemption zone.”

For instance, a former mid-level corporate manager described his choice this way: “I didn’t ‘choose’ university; I simply ‘couldn’t stand’ corporate life anymore. University was the only visible, respectable ‘exit strategy’ within my reach at the time.” On the surface, this quote suggests passive endurance and forced exit, yet the word “decent” reveals an active selection driven by the desire to preserve meaning and dignity amid adversity. This excerpt was coded as “Strategic Retreat: Seeking a Dignified Exit.”

Similarly, a doctoral graduate reflecting on career choices remarked: “I never worked in industry, so it’s not like I ‘escaped.’ I just felt that academia at least preserved the basic notion that ‘evenings and weekends are mine.’ It’s not grand ambition–just defending my baseline.” While “hardly an escape” dismisses proactive motivation, phrases like “preserving the most basic notion” and “defensive stance” reveal a forward-looking concern about losing control over future work–life boundaries. This excerpt was coded as “Proactive Defense,” meaning the protection of a minimum acceptable boundary between work and life.

These initial codes (e.g., “strategic retreat,” “anticipatory defense”) captured the patterned tension of “agentic action under constraint.” They provided the analytic basis for focused coding and later category development (e.g., “defensive career choice” and “building psychological buffers”). Across open coding, 422 initial codes were generated; selected illustrative examples are presented in Table 2.

**TABLE 2:** Open coding (partial).

Data source	Excerpt (example)	Initial code (concept)
Enterprise transitioners	“When I resigned, my boss said I was ‘lying flat.’ I thought: I’m not lying flat–I’m switching to a more sustainable way of standing. At a university, my heartbeat is at least normal.”	Rejecting and reframing the “lying flat” label; pursuing sustainability as a survival stance; prioritizing psychosomatic health
Enterprise transitioners	“I was tired of being a replaceable ‘screw.’ At a university, even if I’m just a small ‘pushpin,’ I can decide where to pin myself on the map of knowledge and leave a mark.”	Identity metaphor shift (from “screw” to “pushpin”); desire for autonomy and leaving an imprint
New-generation doctoral-stage participants	“I’ve never worked in industry, but during internships my leader’s state was the template–everything revolved around ‘output’ and ‘metrics.’ I sensed that ‘who I am’ would be overwritten by ‘what I produced.’ In university interviews, when I said I was ‘long-term curious’ about a theoretical problem, a professor’s eyes lit up. That made me believe this place allows–and even rewards–being unfinished and purely curious.”	Anticipating and resisting instrumental valuation; validating and aligning with imagined academic purity
New-generation doctoral-stage participants	“People call the university a ‘siege,’ but to me, the corporate world is the visible ‘siege.’ I chose the university because its walls seem lower, leaving the possibility of climbing over. So in my application materials, I deliberately downplayed high-output internships and instead described an experiment that failed three times during my Ph.D. I wanted future colleagues to know what kind of researcher I am (resilient, curious).”	Defensive choice via risk comparison; strategic narrative reconstruction oriented to academic identity
Netnographic text	(Blog post titled “From ‘Graduating’ Big Tech to ‘Enrolling’ in a University”) “Today I completed onboarding and received my university staff card. It doesn’t connect to a cold access-control system like a work badge; it opens the library, the gym, and the lakeside running track. Suddenly I felt the card was not exchanging my labor, but granting access to a way of life.”	Symbolic shift from “access tool” to “life-access card”; seeking an integrated work–life form

The excerpts were selected to typify a core analytic pattern: participants’ accounts recurrently displayed agentic action framed as passivity, where apparent endurance or avoidance contained strategic defense, meaning reconstruction, and future-oriented planning. This analytic lens informed subsequent category refinement, including “defensive career choice” and “constructing psychological buffers.”

##### 3.3.1.2 Axial coding

After generating a large set of dispersed initial codes, the analysis proceeded to axial coding. The purpose of this stage was to use constant comparison to cluster conceptually similar codes, develop more abstract and explanatory core categories, and specify each category’s properties and dimensions. This shift marked a move from descriptive labeling to analytic elaboration of the internal structure and variation of concepts.

Through iterative comparison and refinement of the 422 initial codes, four core categories (A–D) were distilled. Table 3 summarizes the subcategories nested within each axial category and uses a consistent analytic template (type, defining features, intensity, and key sources) to articulate category properties and internal variation, thereby preparing the ground for mapping inter-category relationships and subsequent theoretical integration.

**TABLE 3:** Axial coding: core categories (A–D).

Axial category	Subcategories	Properties and dimensions	Relational chain
A. Alienation and exhaustion of career value	KPI-ization of value; instrumentalized self; calculable exhaustion	Types: cognitive distortion; emotional depletion; psychosomatic burnout. Defining feature: personal value and work meaning are defined–and consumed–by external performance systems. Intensity: chronic strain → profound burnout. Key sources: quantified evaluation, hyper-competitive culture, loss of intrinsic meaning.	Performance pressure → value alienation → burnout and exit intention
B. Awakening of psychological safety needs	Seeking breathing space; bottom-line defense; anticipatory fear	Types: restorative safety needs (repair after exit) vs. preventive safety needs (avoid future risk). Defining feature: desire to regain controllability and protect basic life order. Intensity: vague unease → decisive driver. Key sources: response to accumulated exhaustion; anticipation of risks in marketized career paths.	External pressure → internalized safety needs → orientation toward a protective environment
C. Symbolic construction of higher education institutions as an ideal “sanctuary”	Imagined institutional protection; reclaiming time sovereignty; “access to a way of life”	Types: institutional shelter; temporal autonomy; value purity; social belonging. Defining feature: positive cognitive–affective projection combining objective features with subjective expectations. Intensity: alternative option → primary, idealized goal. Key sources: institutional arrangements, subjective needs, and cultural imagery of the “ivory tower.”	Safety needs → projected onto university attributes → university constructed as a symbolic target
D. Strategic identity-transition actions	Identity metaphor shifts; switching impression management; “dignified exit” narratives	Types: internal cognitive redefinition vs. external narrative management. Defining feature: agentic efforts to secure psychological and social legitimacy for field transition. Intensity: private reflection → public behavioral/narrative change. Key sources: managing role conflict; seeking social recognition; self-persuasion.	Cognitive blueprint → identity/narrative work → enacted transition across career fields

##### 3.3.1.3 Selective coding

Building on the clarified category meanings from axial coding, selective coding aimed to achieve final theoretical integration and refinement. The central task was to identify a core category that organizes the theoretical storyline and to explicate how it links and accounts for the other categories.

Through systematic comparison and theoretical abstraction, “constructing a psychological exemption zone” was identified as the core category that best explains the phenomenon. As shown in Table 4, this is not an additional standalone category; rather, it functions as an integrative storyline that dynamically links the full sequence: (A) pressure/risk perception → (B) motivational transformation → (C) goal projection → (D) strategic enactment. In simpler terms, participants first experienced or anticipated psychological risks in marketized workplaces, then looked for a work setting that seemed more protective and sustainable. Here, “constructing a psychological exemption zone” refers to the process through which participants came to see universities as settings that could offer protection, recovery, and greater control over work–life boundaries.

**TABLE 4:** Selective coding: integrative logic of the core category.

Logical dimension	Core meaning and interaction	Contribution to constructing a “psychological exemption zone”
1. Pressure and risk perception	Categories A (value alienation/exhaustion) and B (psychological safety needs) jointly provide the motivational basis for shifting from endurance to change.	Explains the fundamental rationale–why construction becomes necessary: to withdraw from systemic psychological risk.
2. Defensive and constructive choice	Categories B and C: individuals do not merely “choose” passively; they actively translate perceived university attributes into a needs-fulfilling blueprint.	Defines what is constructed: a concrete cognitive target through which abstract needs become actionable.
3. Psychological and career-boundary reconstruction	Categories C and D: the key execution link that converts internal motives and external appraisals into realized transition through cognitive and narrative strategies.	Clarifies how construction is implemented: boundary work and identity/narrative enactment enable psychological migration.
Integrative storyline	The core category integrates the full process: B emerges in response to A; C is constructed because of B; D is enacted to reach C, ultimately aiming at protected autonomy.	Produces a coherent explanatory model: (A) pressure perception → (B) motivational transformation → (C) goal projection → (D) strategic enactment → psychological migration.

#### 3.3.2 Achieving theoretical saturation

Theoretical saturation served as the primary criterion for determining when to stop sampling. In this study, using constant comparison and iterative analysis, saturation was judged to have been reached when analysis of interviews with five additional participants (three transitioners and two new-generation doctoral-stage participants) yielded no new properties, dimensions, or relationships relevant to the core category, “constructing a psychological exemption zone” (Lincoln and Guba, 1985; Naeem et al., 2024; Rahimi and Khatooni, 2024; Squire et al., 2024). At this point, additional data primarily reiterated and substantiated existing categories without generating new theoretical insights; therefore, further sampling and data collection were halted.

#### 3.3.3 Strategies for trustworthiness

To strengthen the study’s trustworthiness, the criteria proposed by Lincoln and Guba (1985), (Johnson et al., 2020; McGill et al., 2023) were followed and the following strategies were implemented.

##### 3.3.3.1 Credibility

Credibility was enhanced through multiple procedures. First, prolonged engagement was undertaken: data collection spanned 7 months, and some participants were interviewed more than once (Staller, 2021). Second, peer debriefing was conducted through regular discussion of coding decisions and preliminary interpretations with two external experts in education and sociology; their feedback helped challenge assumptions and refine categories. Third, member checking was conducted by sharing preliminary analytic summaries with eight participants (four from each group) to assess whether interpretations reflected their experiences and intended meanings; wording was revised based on their feedback.

##### 3.3.3.2 Transferability

Thick description of the research context, participant characteristics, data-collection settings, and analytic procedures was provided, enabling readers to judge the applicability of findings to other contexts. Maximum variation sampling across gender, region, discipline, and destination institution type was also used for both groups, increasing the breadth of empirical variation and the potential transferability of the findings (Schlunegger et al., 2024). However, given the sample size and the study’s focus on shared psychological processes rather than comparative subgroup analysis, systematic comparisons across gender, discipline, or region were not conducted. Future research with larger samples could explore whether these dimensions shape the construction of the “psychological exemption zone.”

##### 3.3.3.3 Dependability

A comprehensive audit trail was maintained, including raw data, transcripts, coding records, analytic memos, iterative model versions, and an analytic decision log. This documentation captures the inferential path from data to conclusions and supports external review and traceability, thereby enhancing dependability of the analytic process.

##### 3.3.3.4 Confirmability

Triangulation was used to ensure that findings were grounded in multiple data sources. Specifically, interview data, online texts, and personal documents were cross-compared; when convergent evidence supported the same interpretation across sources, confidence in that interpretation increased (Gioia et al., 2013). In addition, ongoing team reflexive discussions (see Section “3.1 Research design”) helped mitigate individual bias and supported a data-driven, co-constructed analytic account.

**FIGURE 1:**
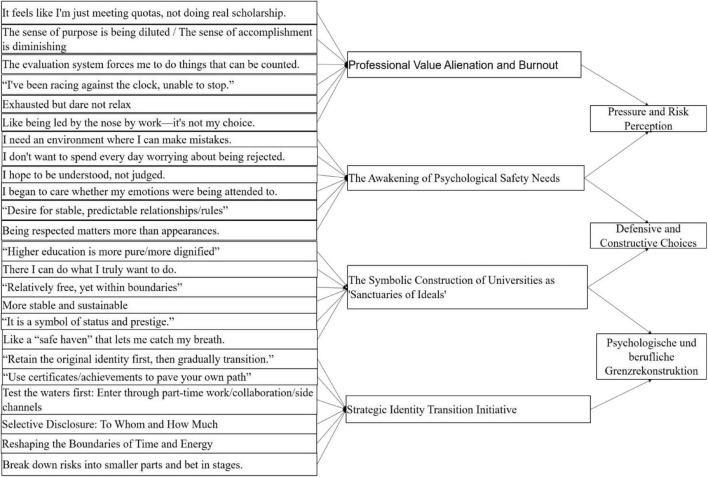
Data encoding structure.

To make the analytic progression transparent, Figure 1 presents the coding structure through which core theoretical constructs were derived from the interview data. Following Gioia et al. (2021), the figure depicts the analytic move from first-order concepts (informant-centric terms) to second-order themes (researcher-centric interpretation), culminating in three aggregate dimensions–“pressure and risk perception,” “defensive and constructive choice,” and “psychological and career boundary reconstruction” (De Vos et al., 2020). We follow constructivist grounded theory as the analytic logic; the Gioia-style figure is used only as a visualization format to improve transparency of the data structure. This data structure not only demonstrates empirical richness but also substantiates the grounding and rigor of the resulting theoretical model.

### 3.4 Ethical considerations

This study was conducted in accordance with the ethical principles of the Declaration of Helsinki. A total of 47 human participants (25 enterprise transitioners and 22 new-generation doctoral-stage participants) took part in semi-structured interviews. All participants provided written informed consent before the interviews and were informed of their rights, including the right to withdraw at any time and to decline to answer any question. Interview data were anonymized at the point of transcription, and participants were assigned numeric identifiers. Any potentially identifying information was removed from transcripts and is not reported in any research outputs. Audio recordings and transcripts were stored securely in encrypted form, with access restricted to the research team.

Through the iterative analytic procedures of constructivist grounded theory, the study developed its central theoretical output: a dynamic model of constructing a “psychological exemption zone.” This model serves as the analytic framework for the next chapter, guiding the organization and presentation of the empirical findings.

### 3.5 Methodological reflexivity and analytic positioning

In addition to the reflexive practices described in Section “3.1 Research design,” this section further clarifies the study’s analytic positioning to address the relationship between prior theoretical knowledge and emergent categories.

It should be acknowledged that the research team entered the field with prior knowledge of self-determination theory, psychological safety, and burnout research. These served as “sensitizing concepts” to orient initial observation and interview design, but they were not imposed deductively (Bakker et al., 2023; Charmaz, 2014; Edmondson and Bransby, 2023; Ryan and Deci, 2020).

The analytic process followed the iterative logic of constructivist grounded theory (Charmaz, 2014), including line-by-line open coding, constant comparison, and theoretical sampling (Foley et al., 2021). Critically, systematic engagement with the career-decision literature was suspended until after open coding had been completed and the core categories (e.g., “strategic retreat,” “anticipatory defense,” “boundary controllability”) had emerged from the data. Only then was the literature revisited to position and refine the emergent constructs (Akkermans et al., 2024; De Vos et al., 2020; Hirschi and Koen, 2021; Sullivan and Al Ariss, 2021).

Thus, the overall strategy is best characterized as abductive–moving iteratively between data and existing theory to generate novel conceptual insight, rather than claiming pure, theory-free induction. This reflexivity statement clarifies that the findings are grounded in participants’ lived experiences, not forced into a pre-existing theoretical mold.

## 4 The analytical framework

### 4.1 A dynamic model of constructing a “psychological exemption zone”

**FIGURE 2:**
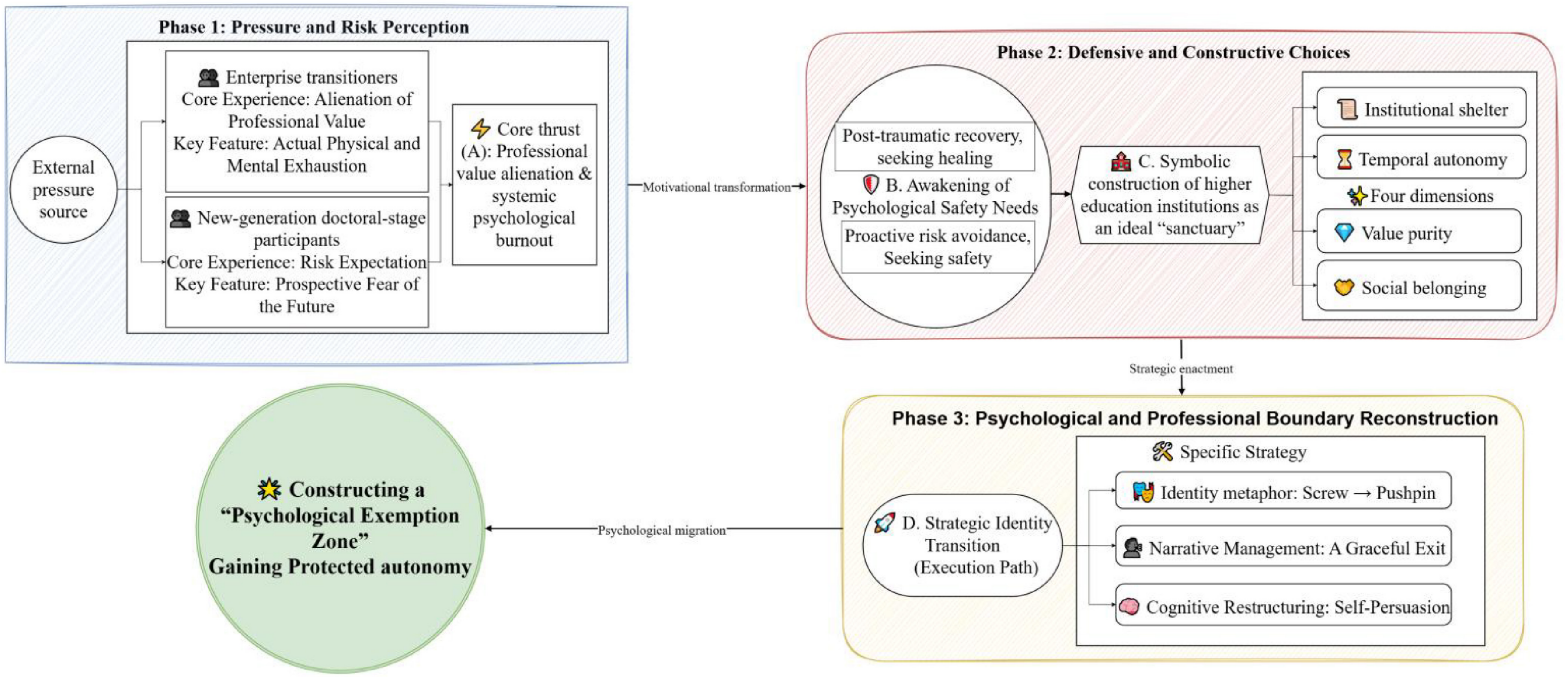
A dynamic model of constructing a “psychological exemption zone.”

Building on the grounded-theory analysis, a three-stage dynamic model of constructing a “psychological exemption zone” was developed to explain the core psychological mechanism underlying movement from a “performance competition arena” to a “psychological exemption zone”: pressure/risk perception → defensive/constructive choice → psychological and career boundary reconstruction. The model is driven by an internal, processual storyline: pressure perception → motivational transformation → goal projection → strategic enactment → psychological migration. Put simply, it explains how participants come to perceive marketized workplaces as risky, imagine universities as safer settings, and then use identity, narrative, and boundary work to support the transition. This chapter is organized around the model. The model is presented in Figure 2.

### 4.2 Three stages and five process steps of the model

The grounded-theory–derived model (Figure 2) delineates three progressive stages in the shift from a “performance competition arena” to a “psychological exemption zone,” and it specifies the internal motivational process that drives this movement. This section explicates the model’s key components to clarify its theoretical logic and explanatory pathway.

#### 4.2.1 Stage 1: pressure/risk perception

This stage constitutes the model’s psychological starting point. At this stage, participants begin to interpret the marketized career environment–the “performance competition arena”–as a source of psychological risk rather than merely as a demanding workplace. For enterprise transitioners, this perception is grounded in direct experience of prior strain and is expressed as “no longer being able to endure,” following prolonged value alienation and emotional depletion. For new-generation doctoral-stage participants, it arises from anticipatory anxiety about future risks and is articulated as an effort to protect a minimum acceptable level of control over work, life, and meaning. Despite these different origins, both groups come to recognize systemic psychological risks and to construct the “performance competition arena” as a threatening space that warrants active defense or exit.

#### 4.2.2 Stage 2: defensive/constructive choice

This stage marks the point at which participants move from enduring pressure to looking for an alternative. “Defensive” refers to the primacy of harm avoidance and safety seeking. “Constructive” indicates that individuals do not select alternatives randomly or resignantly; instead, they connect their psychological needs with specific features they associate with universities, such as secure positions, flexible time arrangements, and a relatively “pure” academic mission. Through this process, the university is constructed as a “psychological exemption zone”–a work setting expected to provide protected autonomy and more predictable meaning. This constructive work marks a motivational reorientation from “where to escape” to “where to go.”

#### 4.2.3 Stage 3: psychological and career boundary reconstruction

This stage is enacted through a set of strategic identity-transition actions: (a) internal identity reconstruction, redefining the self at the cognitive level (e.g., shifting from a “corporate screw” to an “academic pushpin” as a guiding metaphor); (b) external narrative management, mobilizing particular discourses in social interaction, such as “seeking a dignified exit” and “bottom-line defense,” coded in this study as the protection of a minimum acceptable work–life boundary, to legitimate the choice and secure understanding and recognition; and (c) work–life boundary reconfiguration, reallocating time and emotional investment to rebalance work and life, enabling a shift from a “competition rhythm” to a “shelter rhythm.”

#### 4.2.4 The five-step motivational process

Across the three stages runs a continuous internal storyline composed of five linked steps. First, through pressure perception, individuals identify the “performance competition arena” as a source of systemic psychological risk. Second, through motivational transformation, these risks make psychological safety needs more salient. Third, through goal projection, individuals construct the university as an idealized “psychological exemption zone” that may meet these needs. Fourth, through strategic enactment, they use cognitive reframing, narrative management, and behavioral adjustment to support the transition. Finally, through psychological migration, they begin to feel that they have moved from a competitive and depleting work world into a more protective and meaningful one.

## 5 Results

### 5.1 Pressure perception

Before presenting the findings on pressure perception, several related but distinct concepts used by participants to describe their negative work experiences are clarified. In this study:

- Value alienation: the perception that one’s work serves external, quantifiable targets (e.g., KPIs) rather than intrinsic or professional standards.
- Chronic exhaustion: sustained physical and emotional depletion resulting from long working hours and high cognitive load.
- Burnout: a clinically informed syndrome characterized by emotional exhaustion, depersonalization, and reduced personal accomplishment (Maslach et al.).
- Loss of meaning: the subjective sense that work no longer provides purpose or coherence.
- Anticipatory insecurity: future-oriented anxiety about losing control over one’s life trajectory, even without direct negative experiences.

These dimensions often co-occur in participants’ narratives, but they are analytically distinguishable. Section “5.1 Pressure perception” presents them as three escalating subthemes (alienation → exhaustion → insecurity) rather than synonyms, and redundant restatement across subthemes was carefully avoided.

#### 5.1.1 Alienation of career value

Participants repeatedly described a profound sense of self-instrumentalization: their value was no longer tied to expertise or intrinsic contribution but reduced to quantifiable KPIs. A former internet product manager (P03) noted, “My title was ‘senior manager,’ but I felt like a ‘senior number porter.’ Every idea ultimately served that blinking, cold DAU figure.” This KPI-ization stripped work of meaning. A participant who transitioned from finance (P08) reflected, “I once thought I was improving economic efficiency through capital allocation. Later I realized I was serving one earn-out agreement after another and short-term returns. My professional judgment often yielded to the boss’s ‘intuition’ and market ‘noise.”’ For the new-generation group, alienation was frequently articulated as anticipated fear based on observation. A doctoral graduate (S05) said, “During internships, the core skill wasn’t expertise but ‘packaging updates’ and ‘claiming credit while avoiding blame.’ It made me wonder whether my theories would eventually be used only to make prettier slides.”

#### 5.1.2 Systemic psychological exhaustion

Building on value alienation, the “arena” generated pervasive psychosomatic exhaustion. Transitioners described chronic high arousal. A former manufacturing project manager (P12) recalled, “I was on standby 24/7; even in dreams I saw project milestones. It wasn’t ‘996’ but ‘007’–always mentally prepared to work from 0:00 to 0:00. My health report went from all blue to all red in just 3 years.” Participants also emphasized affective “dehumanization.” A former sales director (P17) admitted, “I lost the ability to speak gently–only commands and blame at work, only impatience at home. That environment felt like a high-pressure chamber that squeezed out everything soft, leaving only competition and defensive instinct.” Netnographic materials echoed this atmosphere through recurring metaphors such as “the battery is drained” and “energy has been squeezed dry,” capturing a shared emotional climate.

#### 5.1.3 Anticipated risk and insecurity

Whereas transitioners’ accounts carried a “rear-view mirror” perspective grounded in prior strain, the new-generation group expressed pronounced prospective insecurity. Their pressure perception was less about workload intensity *per se* and more about the fear of losing control over life as a whole. A STEM doctoral student (S11) observed, “Seniors who joined big-tech firms earn a lot, but life itself seems to disappear. I fear that state–high income, yet no time to enjoy it, no energy for relationships, and no room for hobbies. If high pay consumes all of one’s time and emotional energy, it feels like another form of deprivation.” Another doctoral student in the humanities/social sciences (S19) articulated dual concerns about replaceability and meaning: “In a company, my background may be reduced to ‘good writing’–a screw that could be replaced by AI or younger graduates. More importantly, I can’t imagine getting excited about click-through rates and conversion. That feels far from what I consider ‘value.”’ Such insecurity led many to pre-define corporations as a risk source to be avoided even before entry.

### 5.2 Awakening of psychological safety needs and the symbolic construction of the university

When pressure perception crossed a subjective threshold, the need to seek shelter moved from background to foreground and became a decision-driving force. The university was no longer treated as merely one employment option; it was actively constructed as an idealized “psychological exemption zone.” That is, participants came to see the university as a work setting that might protect them from marketized performance risks and help them recover, feel more predictable, and control the boundaries between work and life. From a place-based perspective, this construction also reflects evaluations of the work setting through salient cues and perceived affordances that signal restorability, predictability, and boundary controllability.

#### 5.2.1 From “escape” to “shelter seeking”

Participants’ dominant motive was not simple avoidance (“escaping”) but a more agentic orientation toward seeking shelter. The difference is subtle yet consequential: the former emphasizes what one leaves behind, whereas the latter clarifies where one is going and why. A transitioner (P22) captured this shift: “At first I only wanted to ‘run’–to leave the suffocating environment. But as I searched for a way out, it became clear: I wasn’t running to just anywhere. I was looking for a place where I could breathe, repair myself, and live with dignity. The image of the university gradually emerged from many vague options and became unmistakably clear.” This shift signaled activated agency–from passive endurance toward actively planning a safer future. In environmental terms, shelter seeking entailed selecting a setting expected to reduce chronic strain and support psychological recovery.

#### 5.2.2 Four dimensions of the university as a “psychological exemption zone”

Across accounts, participants constructed the university as a symbolic space that could meet core psychological safety needs through four interrelated dimensions.

The first dimension through which participants constructed the university as a “psychological exemption zone” concerns institutional shelter, particularly security and stability. Tenure-like employment arrangements were widely imagined as a breakwater against market volatility–a partially de-commodified career state that exempts individuals from immediate exchange and elimination logics. A participant leaving a start-up (P05) stated, “After the euphoria of funding rounds and the chill of layoffs, I craved certainty. A university position is like ballast in a career: it may not guarantee how high you fly, but it helps keep you from capsizing.” In this sense, institutional shelter foregrounded environmental predictability as a stress-buffering resource.

The second dimension concerns time sovereignty, understood here as temporal autonomy and clearer work–life boundaries. University work was expected to provide greater discretion over one’s time, offering an explicit counter to the limitless time demands associated with the “arena.” A new-generation participant (S02) noted, “What I value most is the winter/summer breaks and relatively flexible on-site requirements. It means my time comes in whole blocks that I can plan and stitch together. For once, my life rhythm could align with my internal clock.” In this sense, time sovereignty functioned as a form of boundary control, enabling recovery through more stable daily and seasonal rhythms.

The third dimension concerns the purity of meaning production, particularly through teaching and research. Participants endowed teaching and research with intrinsic value and treated them as an antidote to alienation. They expected evaluation to shift, at least partly, from external KPIs back toward knowledge creation and student development. A transitioner (P14) reflected, “In projects we always said we were ‘creating value,’ but it ultimately belonged to clients or shareholders. At a university, when I face students, I know the knowledge I pass on and the thinking I provoke directly shapes people. The value chain is shorter–and feels more real.” In this sense, meaning was enacted through place-based practices, such as teaching and research settings, that supported sustained attention and slower cognition.

The fourth dimension concerns community belonging, especially academic atmosphere and peer recognition. Participants imagined the university as an academic community linked by shared interests and a common discourse. Compared with competitive corporate relationships, they anticipated more supportive ties grounded in professional recognition. A doctoral graduate (S15) said, “I long to discuss ‘this question is interesting,’ not ‘this project is profitable.’ Even when there is competition (e.g., grant applications), it happens within recognized academic rules. That kind of rule-based competition feels safer than the ambiguous ‘connections’ and ‘factional alignment’ in companies.” In this sense, belonging was experienced as a socio-spatial climate that reduced evaluative threat and supported psychological safety.

### 5.3 Strategic identity transition and psychological migration

After projecting the university as a “psychological exemption zone,” individuals needed to translate this cognitive blueprint into enacted transition through strategic actions, culminating in subjective psychological migration.

To inhabit a new role, participants engaged in identity reconstruction by actively redefining the self. A recurring metaphor was the shift from being a corporate “screw” to becoming an academic “pushpin.” As one participant (P10) explained, “A screw is fully passive–its position and function are decided by the machine, and it’s homogeneous and replaceable. A pushpin may be small, but it can choose where to pin itself on the map of knowledge and leave a mark that also locates it. I chose the university because I want to be a pushpin with my own coordinates.” This metaphor marked an identity shift from an instrumental self toward a more agentic self.

Participants also engaged in narrative management when facing questions or skepticism from former colleagues, family members, or the broader public. They mobilized discursive strategies to legitimize their move and to present it as a meaningful and rational choice. For transitioners, “dignified exit” narratives emphasized strategic choice rather than failure, as reflected in statements such as “I wasn’t eliminated; I chose a more sustainable way of living.” For the new-generation group, narratives of baseline protection, or what we coded as “bottom-line defense,” and “risk avoidance” framed the move as rational prevention based on long-term calculation, as illustrated by the statement “I’m not afraid to venture out; I refuse to gamble my entire life on an uncertain future.” These narratives functioned not only as external accounts but also as tools of self-persuasion that reinforced the perceived rationality of the decision.

After entering universities, participants redrew their psychological boundaries by deliberately practicing boundary work to rebalance work and life. This involved actions such as limiting work contact on weekends, resuming hobbies that had been abandoned under overload, and investing more time in family and intimate relationships. A newly appointed young faculty member (S08) shared, “I deliberately mute my work phone after 10 p.m. and keep at least 1 day on the weekend completely laptop-free. These ‘rules’ were impossible in companies, but now they are rituals that protect my personal time and space. In these small moments of control, I feel the self that was once submerged by work being gradually recovered.”

### 5.4 Comparative analysis between the two groups

Although both groups shared the core motive of constructing the university as a “psychological exemption zone,” they differed systematically in pathways and narrative emphases; these differences ultimately converged to reinforce the same construction.

For transitioners, reverse mobility followed a post-strain repair pathway. Their moves were driven by accumulated negative experiences, including health deterioration, profound meaning depletion, and relational rupture. Their accounts foregrounded “past suffering” with strong affect, and their construction of the university therefore emphasized its restorative function: a place to recover from exhaustion, rebuild order, and regain meaning. In this pathway, the move was often narrated as urgent and necessity-driven.

For new-generation doctoral-stage participants, reverse mobility followed an anticipatory risk-avoidance pathway. Unlike transitioners, this group more often acted on imagined and projected risks rather than firsthand damage. Their narratives were more analytic and comparative, frequently introduced through observation and inference, such as “I heard,” “I saw,” or “I suspect.” Accordingly, their construction of the university highlighted prevention: it was imagined as a “safe house” or strategic platform that could help pre-empt foreseeable risks, including loss of life direction and a diminished sense of meaning. In this pathway, the move was framed as rational planning under constrained options, with anxiety oriented toward the future.

Despite their divergent pathways, the two groups jointly contributed to a synergistic construction of a shared social imaginary in which the university was understood as a “psychological exemption zone.” Transitioners’ detailed repair narratives provided vivid and credible anchors for the new-generation group’s risk expectations. Conversely, the new-generation group’s widespread avoidance choices socially validated what might otherwise appear to be a non-mainstream move among transitioners. Together, these two groups participated in an ongoing dialogue about career security and life meaning, through which the symbolic equation of “university = psychological exemption zone” extended beyond individual experience and became a shared psychological schema among segments of young, knowledge-oriented populations.

## 6 Discussion

### 6.1 Positioning the findings within existing theory

The findings both corroborate and refine established perspectives, while also extending conventional explanatory approaches by foregrounding psychological processes in career mobility.

#### 6.1.1 The distinctiveness of the “psychological exemption zone” construct

The “psychological exemption zone” shares conceptual space with established frameworks such as psychological safety, self-determination theory, and decent work. However, it is not merely a relabeling of these ideas. Its distinctiveness lies in its focus on how individuals evaluate an entire work setting as more or less protective.

Psychological safety usually refers to whether people feel safe to speak up, ask for help, or take interpersonal risks within a team or organization. By contrast, the “psychological exemption zone” focuses on how participants came to view certain university settings as safer alternatives to marketized workplaces. It captures their belief that these settings may reduce exposure to KPI-driven value alienation, chronic exhaustion, and anticipatory insecurity.

Similarly, self-determination theory explains how autonomy, competence, and relatedness support motivation. The proposed concept does not replace this explanation. Instead, it shows how participants connect these needs to concrete features of universities, such as institutional shelter, time sovereignty, meaningful work, and community belonging. In relation to decent work and sustainable careers, the concept also shifts attention from objective working conditions to how people imagine, compare, and emotionally invest in a future work setting before they enter it. Thus, the “psychological exemption zone” should be understood as a setting-level concept that explains why universities come to be perceived as spaces of recovery and protection, rather than only as employers.

#### 6.1.2 Extensions of existing theories

This study offers a contextualized Chinese case that illustrates how self-determination theory can help explain career transitions. Exiting the corporate “performance competition arena” can be understood as a response to the sustained frustration of autonomy, competence, and relatedness. The university was constructed as a “psychological exemption zone” because participants perceived it as more able to support these needs. For example, time sovereignty and institutional shelter were linked to the desire for greater control over work–life rhythms, while teaching, research, and academic community were linked to expectations of competence and belonging. Importantly, participants did not passively wait for their needs to be met; through symbolic construction and strategic identity transition, they actively interpreted university features as resources for recovery, meaning, and boundary control.

The findings also add micro-level psychological detail to risk-society accounts of late-modern uncertainty. Macro-level risks, such as “996” work culture and “involution” discourse, were internalized as concrete fears of value alienation, systemic exhaustion, and future loss of life control. In this context, “reverse mobility” functioned as a defensive career strategy rather than a simple occupational shift. Participants did not only change jobs; they tried to reduce their exposure to work environments they perceived as chronically risky. They therefore sought universities, imagined as offering institutional shelter and predictable meaning, to mitigate systemic psychological risks in marketized sectors. This finding shows how perceived features of work settings, such as rhythm stability and boundary controllability, become part of individuals’ defensive coping under uncertainty. The findings further move the analysis of reverse mobility beyond economic rationality by showing how psychological safety and career sustainability shape mobility evaluations. Mobility decisions were not reducible to utility maximization around pay, benefits, or headcount security; rather, participants’ accounts suggest that these decisions also reflected efforts to protect health and well-being through psychological-safety seeking, risk reduction, and recovery.

From a place-based view, such evaluations can be understood as setting-seeking aimed at accessing restorative affordances and boundary-controllable environments. Specifically, for transitioners, the university was constructed as a setting for restoration after accumulated depletion; for the new-generation group, it was constructed as a preventive option to reduce exposure to foreseeable psychological risks (Section “5.4 Comparative analysis between the two groups”). These patterns resonate with the sustainable careers perspective, which argues that career decisions increasingly involve trade-offs among productivity, health, and well-being. Even amid declining relative pay competitiveness and rising “up-or-out” pressures within universities, participants’ narratives indicate that health-, well- being-, and psychological-safety-related concerns could remain salient and, at times, take priority over short-term economic considerations. Future research could further integrate sustainability-oriented career accounts with a work-setting lens that specifies how perceived place cues and environmental affordances shape risk appraisal, recovery expectations, and mobility decisions.

### 6.2 Core theoretical contributions

The model brings into focus a coherent psychological process underlying reverse mobility by integrating insights that have previously been discussed in a more fragmented way.

The findings contribute a process model of psychological transition that integrates pressure/risk perception, defensive/constructive choice, and boundary reconstruction. The model shows how participants move from recognizing pressure, to seeking a safer work setting, and then to rebuilding their identity and boundaries after the transition. This process begins with negative experiences or anticipations in the “arena,” develops into a shelter-seeking motive, and is enacted through identity and narrative work. The final outcome is psychological migration: participants begin to experience themselves as having moved from a competitive work world into a more protective and meaningful one. By clarifying this sequence, the process account may inform research on other forms of defensive career movement under uncertainty.

The findings also show that the university functions not only as a workplace but also as a meaning-laden and affectively invested space. Participants brought to university employment not only job applications but also strong expectations for protected autonomy and predictable meaning. Libraries, laboratories, teaching spaces, and the academic calendar were not treated as neutral organizational features; participants interpreted them as signs of a slower and more humane professional life. Conceptually, the “psychological exemption zone” captures a broader aspiration among knowledge workers for more humane forms of professional life in highly competitive contexts.

### 6.3 Practical implications

For universities, the key implication is the need to align symbolic expectations with organizational realities. Because universities are symbolically invested with idealized expectations as a “psychological exemption zone,” they face corresponding managerial and cultural demands. If pre-tenure reforms reproduce short-term, high-pressure performance competition, universities may fail to deliver the safety and predictability that attract talent in the first place. Therefore, universities may need to re-examine their evaluation systems and strengthen psychologically safe climates that tolerate learning cycles, support experimentation, and enable longer-horizon research. In addition, they can translate “exemption-zone” expectations into concrete work-setting supports. For example, universities can protect more predictable work rhythms through clearer meeting norms, provide quiet spaces for focused work and recovery, and reduce unnecessary monitoring or visibility demands that may increase evaluative pressure.

For firms, the findings highlight the organizational-health costs of hyper-competition. Talent risks stem not only from employee turnover but also from the gradual depletion of employees’ psychological resources. Rather than relying solely on pay incentives and short-cycle quantified competition, firms may benefit from adopting practices that support autonomy, meaning, and psychological safety. Such shifts may reduce exhaustion-driven exits and promote more sustainable engagement. From a work-setting perspective, firms can also help buffer stress and slow depletion by reducing chronic high-strain cues, such as perpetual availability expectations, and by increasing environmental predictability and boundary support, such as clearer off-hours norms.

For individuals, the findings suggest the importance of developing psychological insight in career planning. Treating any sector as an absolute “exemption zone” may be a cognitive shortcut and may increase the risk of expectation–reality mismatch. When making major career decisions, individuals can benefit from clarifying their core needs and values, assessing the target environment realistically, and recognizing that psychological protection also depends on internal resources such as resilience and self-knowledge. They may also evaluate the target work setting in concrete terms, such as rhythm stability, boundary controllability, and socio-spatial climate, rather than relying primarily on sector-level labels or symbolic reputations.

### 6.4 Limitations and reflexivity regarding the proposed model

The dynamic model presented in Figure 2 is an interpretive abstraction derived from the qualitative data, not a universal causal mechanism. The model’s linear presentation–from pressure perception to motivational transformation, goal projection, strategic enactment, and psychological migration–risks oversimplifying the messiness and recursivity of real career decisions. In practice, some participants expressed ambivalence, backtracking, or simultaneous competing motives (e.g., financial pressure versus psychological safety). The model is best read as a stylized narrative template that participants themselves used to make sense of their choices retrospectively, rather than a real-time decision algorithm.

Moreover, the proposed model does not claim that universities always function as “psychological exemption zones” for all individuals. Negative cases were encountered (though not systematically sampled) in which participants who moved to universities later experienced renewed burnout or disappointment due to pre-tenure pressure, heavy teaching loads, or administrative demands. These instances suggest that the “exemption zone” is not a static property of universities but a fragile, context-dependent construction that can dissolve under institutional pressure. Future research with larger and more diverse samples could systematically examine variations across different types of universities (e.g., elite research universities vs. teaching-oriented colleges), disciplines, and career stages, as well as conditions under which universities fail to provide the expected psychological safety.

Finally, readers are encouraged to treat the model as a grounded, transferable heuristic rather than a deterministic law. Qualitative research aims for theoretical transferability, not statistical generalizability. Thick description of the research context and participant characteristics has been provided to enable readers to judge the applicability of the findings to their own settings. Future studies could test, refine, or challenge the model in different cultural and organizational contexts.

## 7 Conclusion

Through an in-depth qualitative investigation of 47 participants–enterprise-to-university “transitioners” and “new-generation” graduate students–this study elucidates the psychological mechanisms underlying the prominent trend of “reverse mobility” among highly skilled individuals in contemporary China. The findings indicate that this choice is not merely a calculation for employment security but a complex psychological process driven by affective motives and meaning-making. In this process, the university is actively imagined, selected, and constructed as an ideal “psychological exemption zone”–a perceived safe space that buffers systemic psychological risks in the marketized “performance competition arena” and enables individuals to regain “protected autonomy” and “predictable meaning.” This imagined “exemption zone” is also grounded in concrete features that participants associate with universities, such as predictability, boundary controllability, and a supportive socio-spatial climate. These features make the university appear to offer greater recovery potential and psychological safety.

The central theoretical contribution of this study is the development of a dynamic model of constructing a “psychological exemption zone.” The model delineates a three-stage psychological pathway–“pressure/risk perception → defensive/constructive choice → psychological and career boundary reconstruction”–and specifies an internal process narrative: “pressure perception → motivational transformation → goal projection → strategic enactment → psychological migration.” In substantive terms, the model shows how individuals first identify psychological risks in marketized workplaces, then imagine universities as safer and more meaningful settings, and finally use identity, narrative, and boundary work to make the transition subjectively and practically possible. Rather than replacing economic and institutional accounts, the model extends them by showing how psychological safety, perceived risk, and work-setting evaluations shape reverse mobility.

Practically, the study highlights that under pervasive uncertainty, psychological safety has become an increasingly central–often implicit–consideration in career decision-making. Both transitioners’ restoration-oriented pursuit following accumulated strain and the new-generation group’s preventive avoidance based on risk anticipation point to a shared aspiration for careers marked by greater controllability, meaning, and belonging. These insights inform how universities and firms can cultivate healthier talent ecosystems and how individuals can approach career planning more reflectively. In practical terms, such efforts include protecting clearer work–life boundaries, reducing chronic high-pressure demands such as perpetual availability expectations, and providing spaces that support focused work and recovery. Overall, the study demonstrates the psychological-safety logic underlying “reverse mobility” and explicates how the university is symbolically constructed as a “psychological exemption zone.” It suggests that in China’s contemporary career landscape, mobility decisions are not only about pay and status but also about sustaining psychological well-being and preserving a meaningful life.

## Data Availability

The datasets presented in this article are not readily available because we have already communicated this data with the investigators and it will not be publicly displayed. Requests to access the datasets should be directed to PD, peitaodu7@163.com.
