# Supplementary material for: From “performance competition arena” to “psychological exemption zone”: psychological safety mechanisms in reverse mobility
Source: Front Psychol. 2026 Jun 16;17:1785539. doi: 10.3389/fpsyg.2026.1785539 (PMC13314783; doi:10.3389/fpsyg.2026.1785539)
Supplement: Supplementary file 1 [file Supplementary_file_1.docx]

# Appendix 1

**Informed Consent Form**

**Study Title: Semi-Structured Interview Protocol: Transition of Doctoral Talents from the “Performance Competition Arena” to the “Psychological Exemption Zone”**

| **Section** | **Content** |
| --- | --- |
| **Invitation to Participate** | You are invited to participate in a research study that explores the psychological experiences, decision-making processes, and identity transitions of doctoral-level individuals who move from corporate or marketized work environments to academic employment, or who choose academia as their primary career pathway.  Before you decide whether to participate, please read the following information carefully. |
| **Purpose of the Study** | The purpose of this study is to understand how highly educated individuals perceive psychological risk, safety, meaning, and boundary control when making major career decisions. In particular, the study examines how universities may be imagined and constructed as psychologically safer or more restorative work settings compared to highly competitive market environments.  This research adopts a qualitative, interview-based approach grounded in psychological and career development perspectives. Your experiences and reflections will help deepen scholarly understanding of career mobility, psychological safety, and well-being. |
| **Procedures** | If you agree to participate, you will be invited to take part in a semi-structured, in-depth interview lasting approximately 60–120 minutes. The interview will focus on topics such as: • Your educational and career background • Experiences or observations of competitive or high-pressure work environments • The process through which you considered or enacted a transition to academia • Your expectations of academic work and perceived psychological differences between sectors • Changes in professional identity, meaning, and work–life boundaries  With your permission, the interview will be audio-recorded to ensure accuracy. The recording will later be transcribed verbatim for analysis. |
| **Voluntary Participation and Right to Withdraw** | Your participation in this study is entirely voluntary.  You may decline to answer any question and may withdraw from the study at any time without penalty or negative consequences. If you choose to withdraw, any data already collected from you will be deleted upon request. |
| **Risks and Discomforts** | The study involves minimal risk.  Some questions may prompt reflection on stressful work experiences or career uncertainty, which could cause mild emotional discomfort. You are free to pause, skip questions, or stop the interview at any time. No deception is involved. |
| **Benefits** | There is no direct material benefit for participation. However, many participants find the interview to be a valuable opportunity for reflection.  More broadly, your participation will contribute to academic knowledge on psychological safety, career sustainability, and well-being among highly educated professionals. |
| **Confidentiality and Anonymity** | All information you provide will be treated as strictly confidential.  • Your name and any identifying details will be removed during transcription. • Pseudonyms or numerical codes will be used in all research materials and publications. • Audio recordings, transcripts, and related documents will be stored securely in encrypted form and accessed only by the research team. • Any quotations used in publications will be fully anonymized to prevent identification. |
| **Use of Data** | The data collected may be used for academic publications, conference presentations, and related scholarly outputs. All use of data will comply with ethical guidelines for research involving human participants. |
| **Ethical Approval** | This study has received ethical approval from an appropriate institutional ethics review committee. The research is conducted in accordance with established ethical standards for psychological and social science research. |
| **Contact Information** | If you have questions about the study or your participation, you may contact the researcher:  Researcher: [Your Name] Affiliation: [Your Institution] Email: [Your Email]  If you have concerns about your rights as a participant, you may contact the relevant ethics review committee. |

**Consent Statement**

By signing below, you confirm that you have read and understood the information above, have had the opportunity to ask questions, and voluntarily agree to participate in this study.

| Participant Name | ______________________________ |
| --- | --- |
| Signature | ______________________________ |
| Date | ______________________________ |

# Appendix 2

# Semi-Structured Interview Protocol: Transition of Doctoral Talents from the “Performance Competition Arena” to the “Psychological Exemption Zone”

## I. Pre-interview Notes (For the Interviewer)

This protocol is designed to support open-ended, narrative interviewing about career transitions from marketized work contexts to academic employment. The metaphors "Performance Competition Arena" and "Psychological Exemption Zone" are used only as sensitizing concepts; participants should be encouraged to describe experiences in their own terms.

**Core objectives:**

- Understand the psychological decision-making process behind doctoral talents’ transition from corporate/market sectors to academia.
- Capture experiential narratives of the "Performance Competition Arena" (corporate/market contexts) and the anticipatory construction of a "Psychological Exemption Zone" (academia).
- Explore strategies and emotional logics involved in professional identity transformation, meaning reconstruction, and boundary management.

**Interviewing principles:**

- Open-endedness: Use “how,” “why,” and “could you describe” prompts; avoid yes/no questions.
- Neutrality: Maintain a non-judgmental stance toward terms like “stress,” “burnout,” or “escape.” Follow the participant’s language.
- Flexibility: Follow the participant’s narrative flow; deviate from the sequence when natural.
- Theoretical sensitivity: Introduce concepts such as psychological safety, boundary controllability, or institutional shelter through gentle probing, without defining them as “correct” answers.

**Participant groups:**

- Enterprise Transitioners: PhD holders who previously worked in marketized firms and have moved to academia.
- Doctoral-stage Participants: Final-year PhD candidates or PhD graduates within 1–2 years post-graduation who are pursuing academic employment.

**Estimated interview length:** 60–90 minutes (adjust flexibly).

## II. Interview Modules and Core Questions

Note: Use the module timing as a guide. Record vivid phrases, metaphors, and moments of emotional intensity, and probe for concrete episodes (who/what/when/where) when needed.

### Module A. Career Context and Turning Points (≈10 minutes)

(Aims: build rapport, gather background, and identify transition catalysts.)

1. Could you briefly walk me through your educational background and key career experiences?
2. Just before you seriously considered moving to academia, how would you describe your state of work/study? What keywords come to mind for that period?
3. For Enterprise Transitioners: When did you start seriously considering leaving the corporate sector for academia? Was there a specific tipping point or event?
4. For Doctoral-stage Participants: When did you start viewing an academic position as your primary post-graduation goal? What reinforced this preference?

### Module B. Experiencing and Narrating the "Performance Competition Arena" (≈15 minutes)

(Aims: elicit rich descriptions of marketized work contexts, pressure perception, and value alienation.)

1. Looking back at your corporate/internship/observation experience, how would you describe the work culture and competitive atmosphere?
2. How was performance defined and measured in that environment? How did those measures shape your daily practices and your sense of self?
3. Did you feel a constant encroachment of work on your personal time, energy, or emotions? Could you describe a memorable example?
4. In that context, how were your expertise, creativity, or intrinsic motivations utilized, supported, or challenged?
5. If you felt pressure or insecurity, where did it come from most often (e.g., metrics, supervisors, peers, clients, uncertainty)? Can you recall an episode?

### Module C. Decision Psychology and Constructing Expectations of Academia (≈20 minutes)

(Core module: focuses on how expectations of a "Psychological Exemption Zone" are constructed.)

1. When the idea of entering academia first emerged, what was the initial appeal? What specific images, experiences, or comparisons drew you in?
2. Compared to your previous environment, in what ways did you expect academia to be different? (Prompts: work rhythm, evaluation criteria, interpersonal dynamics, personal controllability.)
3. Which institutional features of academia (e.g., academic calendar, job security arrangements, research autonomy, governance) were significant in your decision? What did they represent to you psychologically?
4. Did you expect to find a stronger sense of meaning in academic work? How did you imagine teaching or research would provide that meaning?
5. On a psychological level, did you expect academia to be a more safe, restorative, or breathable space? Please describe that expectation in concrete terms.
6. Were there any worries or “costs” you anticipated in choosing academia? How did you weigh them against perceived benefits?

### Module D. Identity Transition, Narrative Management, and Boundary Practices (≈15 minutes)

(Aims: capture psychological and social work undertaken to enact the transition.)

1. How has this career shift changed your professional identity—your sense of who I am? How do you explain this shift to yourself?
2. When facing questions (or skepticism) about this choice from family, friends, or former colleagues, how do you usually explain your decision?
3. To adapt to or realize your expectations of academic life, have you consciously changed your work habits, time allocation, or social patterns? Please give an example.
4. What boundaries do you try to protect now (e.g., time, emotions, relationships, autonomy)? What helps you maintain them?

### Module E. Reality Check and Reflective Summary (≈10 minutes)

(Aims: assess expectation–reality gaps and deepen reflexivity.)

1. Since entering academia (or pursuing it), which expectations have been confirmed? Which aspects differed from your imagination?
2. In hindsight, to what extent do you think the idea of "academia as a psychological exemption zone" holds true? Does academia present its own challenges or new arena-like dynamics?
3. Reflecting on the entire transition, what was the single most central factor driving your choice?
4. Is there anything important we did not discuss that you think is essential to understand your experience?

## III. Interview Closing Statement (Suggested Script)

Thank you for your open and detailed sharing. Your experiences and reflections provide an important perspective for understanding career choices of highly educated talents. This concludes our formal interview. If we quote your narratives in any publications, they will be fully anonymized. We appreciate your time and wish you all the best in your future career path.
